# Papillary thyroid carcinoma within struma ovarii: a case report and literature review

**DOI:** 10.1093/jscr/rjaf067

**Published:** 2025-02-17

**Authors:** Aras J Qaradakhy, Rawa M Ali, Rebaz M Ali, Hadeel A Yasseen, Shvan M Hussein, Hiwa O Abdullah, Shko H Hassan, Harun Amanj Ahmed, Abdulwahid M Salih, Snur Othman, Fahmi H Kakamad

**Affiliations:** Scientific Affairs Department, Smart Health Tower, Madam Mitterrand Street, Sulaymaniyah 46001, Iraq; Radiology Department, Shorsh Teaching Hospital, Shorsh Street, Sulaymaniyah 46001, Iraq; Scientific Affairs Department, Smart Health Tower, Madam Mitterrand Street, Sulaymaniyah 46001, Iraq; Scientific Affairs Department, Smart Health Tower, Madam Mitterrand Street, Sulaymaniyah 46001, Iraq; Hiwa Cancer Hospital, Saiwan Street, Sulaymaniyah 46001, Iraq; Scientific Affairs Department, Smart Health Tower, Madam Mitterrand Street, Sulaymaniyah 46001, Iraq; College of Medicine, University of Sulaimani, Madam Mitterrand Street, Sulaymaniyah 46001, Kurdistan, Iraq; Kscien Organization for Scientific Research (Middle East office), Hamdi Street, Azadi Mall, Sulaymaniyah 46001, Iraq; Scientific Affairs Department, Smart Health Tower, Madam Mitterrand Street, Sulaymaniyah 46001, Iraq; Kscien Organization for Scientific Research (Middle East office), Hamdi Street, Azadi Mall, Sulaymaniyah 46001, Iraq; Scientific Affairs Department, Smart Health Tower, Madam Mitterrand Street, Sulaymaniyah 46001, Iraq; Scientific Affairs Department, Smart Health Tower, Madam Mitterrand Street, Sulaymaniyah 46001, Iraq; Scientific Affairs Department, Smart Health Tower, Madam Mitterrand Street, Sulaymaniyah 46001, Iraq; Scientific Affairs Department, Smart Health Tower, Madam Mitterrand Street, Sulaymaniyah 46001, Iraq; Scientific Affairs Department, Smart Health Tower, Madam Mitterrand Street, Sulaymaniyah 46001, Iraq; College of Medicine, University of Sulaimani, Madam Mitterrand Street, Sulaymaniyah 46001, Kurdistan, Iraq

**Keywords:** ovarian goiter, papillary thyroid carcinoma, struma ovarii, teratomas, thyroid

## Abstract

Struma ovarii (SO) is a rare ovarian teratoma mostly made of thyroid tissue, with papillary thyroid carcinoma (PTC) being even rarer. This report presents a 50-year-old woman with PTC in SO and a normal thyroid. A left ovarian cyst was detected by ultrasound and confirmed by MRI. Surgery included total abdominal hysterectomy with bilateral salpingo-oophorectomy, revealing focal PTC and no thyroid abnormalities. A review of 10 cases showed a median diagnosis age of 46.5 years, with symptoms like abdominal pain and vaginal bleeding. Routine checkups in perimenopausal women are key for early detection, and thyroid evaluation is important in PTC cases within SO.

## Introduction

Struma ovarii (SO) is a rare ovarian teratoma subtype, primarily composed of thyroid tissue (>50%), earning it the term “ovarian goiter” [1]. It can exhibit various thyroid pathologies, both benign and malignant. Malignant features are rare, occurring in <10% of cases, and may include papillary thyroid carcinoma (PTC), its follicular variant, or mixed variants, which share molecular and prognostic similarities with thyroid-origin tumors [2–4]. The cause of PTC within SO is unclear, typically affecting women aged 40–60. It is vital to differentiate primary ovarian PTC from thyroid metastasis due to differing prognoses and treatments [5, 6]. This report presents a case of primary PTC within SO in a patient with a normal thyroid, following CaReL guidelines and peer-reviewed references [7, 8].

## Case presentation

A 50-year-old unmarried woman presented for a routine checkup due to amenorrhea. Ultrasound (U/S) incidentally detected a left ovarian cyst. She had no significant medical, surgical, or family history and was vitally stable with normal physical and lab findings. Pelvic U/S showed a left ovarian cyst (84 × 52 × 55 mm), an anteverted uterus with 8 mm endometrial thickness, no free fluid, and an empty uterine cavity. MRI identified three fibroids (largest 65 × 63 × 57 mm) and a left ovarian cystic-solid lesion (74 × 65 × 48 mm) suggestive of a cystic adenofibroma. No pathological pelvic lymph nodes were observed.

**Figure 1:**
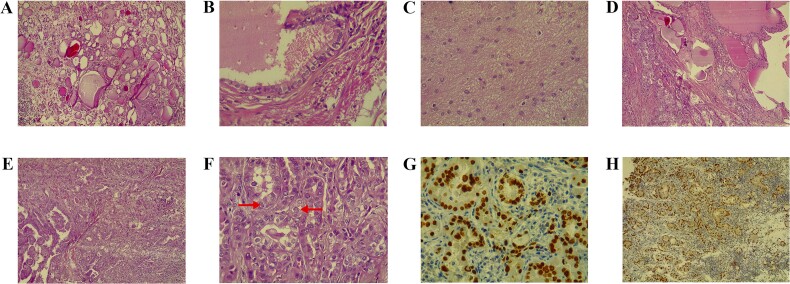
A) The teratoma predominantly comprises benign thyroid tissue (hematoxylin and eosin stain, magnification 100×). B) Higher magnification shows a focal duct-like structure lined by ciliated epithelium (hematoxylin and eosin stain, magnification 400×). C) Another focus demonstrates mature glial tissue (hematoxylin and eosin stain, magnification 40×). D) The section depicts normal thyroid tissue on the right transitioning into papillary thyroid carcinoma on the left (hematoxylin and eosin stain, magnification 100×). E) A small focus of papillary projections with numerous crowded follicular structures, indicative of papillary thyroid carcinoma (hematoxylin and eosin stain, magnification 100×). F) High-power view shows crowded follicular structures with enlarged, optically clear nuclei that show occasional pseudoinclusions (arrows), features characteristic of papillary thyroid carcinoma (hematoxylin and eosin stain, magnification 400×). G) Immunohistochemical stain for TTF1 demonstrating strong, diffuse, nuclear staining in the crowded follicles (diaminobenzidine chromogen, magnification 400×). H) Immunohistochemical stain for PAX-8 showing a strong, diffuse, nuclear staining pattern in the crowded glands (diaminobenzidine chromogen, magnification 100×)

Under general anesthesia, a total abdominal hysterectomy with bilateral salpingo-oophorectomy was performed. Histopathology revealed a 7.5 × 7 × 6 cm mass resembling thyroid tissue. Microscopy showed a cyst lined by ciliated epithelium and various mature elements, including thyroid follicles, glial tissue, and lymphoid follicles. A small invasive glandular area exhibited papillary structures with nuclear features of PTC. Stromal changes included hyalinization, histiocyte aggregation, calcification, and luteinization. Immunostaining showed >90% positivity for PAX8 and TTF1, 50% for CK7, and < 5% for Napsin-A. These findings confirmed PTC arising within SO (Fig. 1).

Postoperative recovery was uneventful. Further evaluation showed euthyroid status and normal thyroid U/S findings.

**Table 1:** The characteristics of the reviewed case reports

**Author/year** **[Reference]**	**Country**	**Age**	**Sex**	**Presentation**	**Radiology**	**Management**	**Histopathology**	**Outcome**
Chiu *et al.* 2020 [1]	Taiwan	47	F	Vaginal spotting	U/S: big cystic mass with central sand-like consistency in left adnexa (Size: 11.61 cm)	TAH + BSO	PTC (12.5 × 8.0 × 4.0 cm)	No recurrence or metastasis
Al Hassan *et al.* 2018 [3]	Qatar	42	F	Lower abdominal pain	U/S: large solid cystic mass in the right adnexa region, reaching the midline (≈6 × 13 cm) with mild vascularity in the solid component. CT & MRI: confirmed the size and solid/ cystic nature of the mass and showed no metastatic lesions, and also deviation of the uterus to the left side.	TAH + BSO + infracolic omentectomy	PTC	No malignancy in the right fallopian tube, uterus, or cervix, and negative lymph nodes.
Winata *et al.* 2023 [5]	Indonesia	50	F	Abdominal pain	U/S: probe-sized hypo-hyperechoic image with a solid part and septa.	TAH + BSO, chemotherapy	PTC (20 × 20 cm)	Complete response and no mass residual
Hmidi *et al.* 2023 [6]	Tunisia	37	F	Incidental finding during cesarean section	NA	Cystectomy	PTC (20 × 20 × 10 mm)	No recurrence 7 years after surgery
Leuștean *et al.* 2022 [10]	Romania	46	F	Incidental finding during routine U/S examination	U/S: large right ovary (57 × 38 mm) with a relatively well-defined cystic image in the periphery (44 × 43 mm). Its size increased after six months. MRI: right adnexal mass (79 × 66 × 72 mm).	TAH + left laparoscopic adnexectomy	Follicular variant of PTC. (90 mm)	No recurrence after 1-year follow-up.
Kabootari *et al.* 2023 [13]	Iran	47	F	Abnormal uterine bleeding	U/S: bilateral adnexal masses (51 × 31 mm in the right ovary and 38 × 29 mm in the left ovary). CT: confirmed left ovarian mass and a mass in the abdominal wall.	TAH + BSO	Mixed papillary and follicular variant of PTC	No recurrence or distant metastasis after six years.
Rahimi *et al.* 2024 [14]	Iran	64	F	Abdominal pain	U/S: a large, heterogeneous mass with an irregular border (43 × 45 mm) in the left ovary. MRI: a large cystic lesion with a solid component in the left ovary, strongly indicating left ovarian cancer.	TAH + BSO	PTC	No distant metastasis
Ioannidis *et al.* 2021 [15]	Greece	65	F	Abdominal pain	U/S: A mass (37 × 27 × 37 mm) was detected in the ovary. CT: same as U/S MRI: indicating dermal cysts. The dimension of the mass was 73 × 40 × 51 mm.	TAH + BSO	Mature cystic teratoma of the right ovary, in which PTC was noted.	No signs of recurrence or any other complications after four months.
Alamdari *et al.* 2018 [16]	Iran	10	F	Palpitation	U/S: a solid mass (113 × 112 × 100 mm) with volume of 670 cc in the right ovary with no ascites.	Unilateral oophorectomy	PTC	Symptom-free following eight months after surgery and iodine therapy
Kim *et al.* 2023 [17]	South Korea	22	F	Abdominal pain	CT and U/S revealed bilateral ovarian cysts (each approximately 10 cm in size) and left ovarian torsion.	Laparoscopic bilateral ovarian cystectomy	PTC	Stable health condition during 10 months after surgery

BSO, bilateral salpingo-oophorectomy. CT, computed tomography. MRI, magnetic resonance imaging. NA, not available. PTC, papillary thyroid carcinoma. TAH, total abdominal hysterectomy. U/S, ultrasound.

## Discussion

Teratoma, the most common ovarian germ cell tumor, is usually benign, with malignant transformation (<2%) often as squamous cell carcinoma (80%) and rarely as thyroid carcinomas (0.1%–0.2%) [6, 9]. SO, where thyroid tissue forms >50% of a teratoma, accounts for 2%–5% of teratomas and 0.5%–1% of ovarian tumors. Malignancy in SO (<10%) is mainly PTC (53%), followed by its follicular variant (41%) and follicular thyroid carcinoma (FTC) (6%) [2, 6, 10]. PTC pathogenesis in SO is unclear, with theories involving germ cells, ectopic thyroid, somatic transformation, hormones, and immunity [6].

Risk factors for PTC include radiation, diabetes, female gender (three times higher risk), obesity, smoking, alcohol, genetic factors, and excessive dietary nitrates and iodine. Thyroid carcinomas often coexist with breast, colon, stomach cancers, and non-Hodgkin lymphoma [11, 12], but thyroid carcinoma linked to ovarian disease is rare [2, 3]. SO, symptoms range from asymptomatic to abdominal pain, palpable masses, ascites, abnormal vaginal bleeding, hyperthyroidism, and Meigs syndrome [4]. Kabootari *et al.* reported a 47-year-old woman with abnormal uterine bleeding, diagnosed with PTC in SO [13]. Al Hassan *et al.* described a 42-year-old with intermittent abdominal pain and regular cycles [3].

In 10 reviewed cases [1, 3, 5, 6, 10, 13–17], the median age at diagnosis was 46.5 years, with abdominal pain and vaginal bleeding as common symptoms (Table 1). This report describes a 50-year-old woman with menstrual cessation and menopausal signs after a routine checkup, with no notable medical history.

SO, on U/S may appear as a heterogeneous, unilocular or multilocular solid mass, or multilocular cystic masses, with “struma pearls” as key features, vascularized on Doppler [3]. MRI can resemble ovarian carcinomas, showing mixed cystic and solid components or adnexal masses with ascites, but lacks specificity for malignant transformation except in cases of rapid tumor growth with irregular borders [10]. U/S was the primary diagnostic tool in the reviewed cases, supplemented by CT and MRI. In this case, U/S and MRI showed a multiloculated cyst with no vascularity, pelvic fluid, or abnormal lymph nodes.

The optimal treatment for malignant SO is debated. Surgical options include bilateral salpingo-oophorectomy with omentectomy and lymph node sampling, often with total abdominal hysterectomy, especially for postmenopausal women [15, 16]. For younger patients, unilateral oophorectomy may be considered [4, 17]. Post-operative histopathology confirms SO by identifying thyroid follicles with patterns like micro- and macro-follicles [3]. In malignant SO, distinguishing primary ovarian tumors from secondary thyroid tumors is essential [5]. Thyroidectomy is recommended for monitoring, though a stable case was reported without thyroidectomy for seven years [6]. PTC in SO shares mutations like BRAF, RET, and RAS with primary thyroid cancers [6]. This case showed mostly benign thyroid tissue with a small area of papillary carcinoma.

When SO is found incidentally, thyroid function tests and neck U/S are key to check for coexisting pathology. PTC within SO generally has a good prognosis [1, 5, 6, 13]. In this case, thyroid function was normal, and neck U/S showed no issues, so the patient was placed on follow-up. A limitation of this report is the lack of radiologic images. Routine checkups, especially in perimenopausal women, are important for detecting silent conditions like SO, and thyroid assessment may be needed in PTC cases to rule out metastasis.
